# Characterization of the accessory protein ClpT1 from *Arabidopsis thaliana*: oligomerization status and interaction with Hsp100 chaperones

**DOI:** 10.1186/s12870-014-0228-0

**Published:** 2014-08-24

**Authors:** Clara V Colombo, Eduardo A Ceccarelli, Germán L Rosano

**Affiliations:** Instituto de Biología Molecular y Celular de Rosario (IBR), CONICET, Facultad de Ciencias Bioquímicas y Farmacéuticas, Universidad Nacional de Rosario, Esmeralda y Ocampo, Rosario, Argentina

**Keywords:** ClpT1, Hsp100 chaperones, ATPase activity, Protein quality control, Accessory protein, *Arabidopsis thaliana*

## Abstract

**Background:**

The caseinolytic protease (Clp) is crucial for chloroplast biogenesis and proteostasis. The *Arabidopsis* Clp consists of two heptameric rings (P and R rings) assembled from nine distinct subunits. Hsp100 chaperones (ClpC1/2 and ClpD) are believed to dock to the axial pores of Clp and then transfer unfolded polypeptides destined to degradation. The adaptor proteins ClpT1 and 2 attach to the protease, apparently blocking the chaperone binding sites. This competition was suggested to regulate Clp activity. Also, monomerization of ClpT1 from dimers in the stroma triggers P and R rings association. So, oligomerization status of ClpT1 seems to control the assembly of the Clp protease.

**Results:**

In this work, ClpT1 was obtained in a recombinant form and purified. In solution, it mostly consists of monomers while dimers represent a small fraction of the population. Enrichment of the dimer fraction could only be achieved by stabilization with a crosslinker reagent. We demonstrate that ClpT1 specifically interacts with the Hsp100 chaperones ClpC2 and ClpD. In addition, ClpT1 stimulates the ATPase activity of ClpD by more than 50% when both are present in a 1:1 molar ratio. Outside this optimal proportion, the stimulatory effect of ClpT1 on the ATPase activity of ClpD declines.

**Conclusions:**

The accessory protein ClpT1 behaves as a monomer in solution. It interacts with the chloroplastic Hsp100 chaperones ClpC2 and ClpD and tightly modulates the ATPase activity of the latter. Our results provide new experimental evidence that may contribute to revise and expand the existing models that were proposed to explain the roles of this poorly understood regulatory protein.

**Electronic supplementary material:**

The online version of this article (doi:10.1186/s12870-014-0228-0) contains supplementary material, which is available to authorized users.

## Background

Protein quality control is an array of cellular mechanisms through which protein homeostasis is monitored and maintained. This process involves the refolding, sequestration, or degradation of misfolded polypeptides, which may be deleterious to the cell due to their propensity to aggregate [1,2]. They arise as byproducts of *de novo* synthesis or are caused by cellular stress, structure-disruptive mutations or simply, structural changes at the end of the protein life cycle [3]. Proteins that are damaged beyond repair or are not longer needed are eliminated through proteolytic degradation. At the heart of this cellular phenomenon are energy-dependent proteases, which are in charge of polypeptide turnover. In general, complete degradation of target polypeptides is carried out by complex multisubunit proteases such as FtsH, the 26S proteasome, and the Clp protease [4,5]. At the molecular level, these proteases form intricate barrel-shaped structures harboring the active sites. The substrate enters the proteolytic chamber through the axial pores and gets subsequently degraded by the action of a peptide bond hydrolyzing serine residue [6–8]. However, many of these proteases do not recognize nor unfold their substrates directly. Rather, they associate with ATP-dependent molecular chaperones that deliver the unfolded target to the degrading machine [9].

Protein turnover in chloroplasts is a highly dynamic process. Phase transition and senescence implicate massive protein degradation [10,11]. In addition, light energy constantly damages photosynthetic proteins [12]. That is why these organelles possess a full arsenal of proteases that keep in check protein homeostasis [10,13]. In particular, the Clp protease is one of the most important proteolytic system in the stroma [14]. In *Arabidopsis thaliana*, it consists of two stacked heptameric rings that define the proteolytic cavity [15]. The rings were named P-ring or R-ring depending of their subunit composition. ClpP3-6 conform the P-ring, while ClpP1 and ClpR1-4 are part of the R-ring [16]. Apparently, substrate recognition, binding and unfolding lie on the chaperone partner, namely ClpC1/2 and ClpD. These Hsp100 chaperones can assemble into hexamers with a molecular mass of 500 – 600 kDa [17] and are believed to dock to the axial pores of the ClpPR core [15]. The fully-competent degrading machine is thus made of a dozen different proteins. The Clp system is also found in bacteria; however, it is much simpler than its plant counterpart in terms of subunit type composition. For example, in *Escherichia coli*, the Clp system is made of the homo-oligomeric ClpP protease, which can associate with the chaperones ClpA or ClpX [18].

Other Clp proteins which may regulate the assembly and function of the Clp system have been found. ClpS is a regulator protein which seems to be the substrate selector for the Clp system in chloroplasts of *A. thaliana* [19]. ClpT1 and ClpT2 are small proteins exclusively found in plants. Initially, they were annotated as nClpC-like proteins, due to their homology to the N-terminus of ClpC. Both were then identified as part of the Clp system by mass spectra analysis of Clp complexes isolated by “colorless native” gel electrophoresis [20]. They were found to associate peripherally to the Clp complex and seem to regulate its assembly [21]. Null mutants in either *clpT1* or *clpT2* do not show noticeable phenotypic changes from the wild type, while the double mutant is seedling lethal [21]. For that reason, a molecular approach is more appropriate to gain further insight into the function of these accessory proteins. Here, we show that one of the ClpT proteins (ClpT1, obtained in a recombinant form) interacts with the chaperone components of the Clp complex (ClpC2 and ClpD) and specifically stimulates the ATPase activity of ClpD. Structurally, recombinant ClpT1 exists mainly as a monomer in solution but can associate into dimers in a small proportion. Our results provide experimental evidence that raises new questions about the role of this poorly understood regulatory protein.

## Results

### Expression and purification of recombinant ClpT1

**Figure 1:**
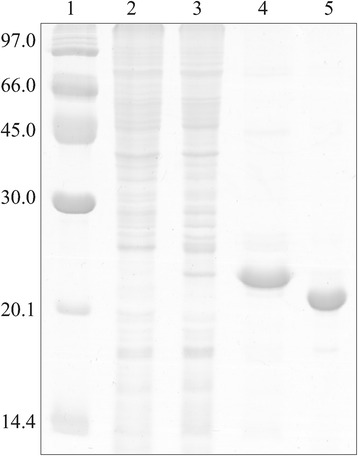
**Purification of recombinant ClpT1.** Expression and purification of the recombinant protein ClpT1 were evaluated by gel electrophoresis and Coomassie staining. Soluble extracts from uninduced and induced cultures were loaded in lanes 2 and 3, respectively. Lane 4 shows the eluted protein after affinity chromatography and lane 5, ClpT1 after the whole purification procedure. Molecular weight markers were loaded on lane 1; their molecular weights are stated on the left.

To produce ClpT1 in a recombinant form, the sequence encoding for the mature protein was cloned into a pET28 expression vector. The mature N terminus was determined using the prediction tool ChloroP [22]. Structure modeling of ClpT1 using SWISS-MODEL showed that the N-terminal end seems to be inaccessible to the solvent (data not shown), so we chose to place the His-tag at the C-terminal end. ClpT1 was expressed from a T7 promoter-based vector in *E. coli* and recovered by immobilized-metal affinity chromatography and size exclusion chromatography (SEC). The C-terminal histidine tag was removed by thrombin digestion. ClpT1 was isolated to >98% purity and its molecular mass corresponded to that of the mature native protein (22 kDa, Figure 1). We also attempted to produce ClpT2 using the same experimental approach. However, during the thrombin digestion step, a fraction of the protein precipitated and the remaining was digested by the protease. Efforts to optimize cleavage conditions were unsuccessful. We chose not to characterize uncleavaged ClpT2 as modifications at the C-terminal end (including adding a histidine tag) may cause artifacts in interaction assays with Hsp100 chaperones, as was seen for ClpA [23].

### Oligomerization status of ClpT1

**Figure 2:**
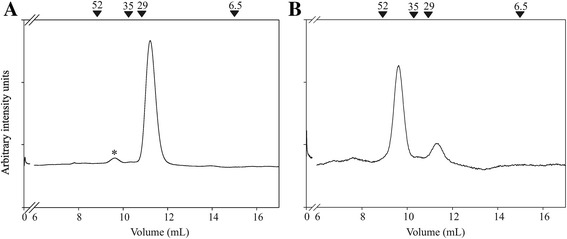
**Oligomerization status of ClpT1. (A)** Elution profile of the purified protein. Arrows above the plot indicate the migration of molecular weight standards. **(B)** The peak corresponding to the dimer in **(A)** (marked with an asterisk) was collected and subjected to a second SEC step. The corresponding elution profile is shown.

A recent report showed that recombinant ClpT1/2 and native ClpTs from stroma extracts assemble into dimers in gradient native gels run for 48 hs [21]. We used the milder SEC technique to analyze the oligomerization status of ClpT1. Two major peaks were detected (Figure 2A). One peak corresponding to a molecular mass of 44 kDa indicates the presence of the dimer species. The other centered at around 22 kDa corresponds to the monomer. These results were confirmed by static light scattering of the eluted samples (data not shown). Peak integration revealed that the peak corresponding to the dimer species represents less than 5% of the total ClpT1 population, which is in contrast with the previous work of Sjögren and Clarke that showed a clear predominance of the dimer species. In fact, monomeric ClpT1 was not detected in that study. The effect of ClpT1 concentration on dimer formation was assayed by injecting a 6-fold concentrated sample and a 10-fold diluted sample into the column, yet the amount of dimer did not change (<5% compared to the monomer species in both cases, data not shown). Also, dimer formation was not induced by changing some environmental conditions in the chromatographic run. We emulated some of the conditions used by Sjögren and Clarke in their experimental setup, for example by changing buffer composition, time of analysis and by using His-tagged ClpT1. Either ClpT1 or ClpT1-(His)_6_ were incubated with 45 mM borate buffer for 1 hr or three days at 4°C and analyzed by SEC. Again, no changes in the ratio of dimer:monomer was seen in any case (data not shown). To rule out possible unspecific interactions of ClpT1 with the dextran resin that could have altered a proper molecular mass determination, ClpT1 was incubated for 1 hour in 750 mM NaCl or 1 mM free dextran and subjected to SEC under these conditions, but the positions of the peaks remained unaltered (data not shown). It should be noted that, once formed, the dimers seem to be stable. Collecting the small dimer peak and reinjecting it into the column showed that this time, the dimer peak represented more than 92% of the total ClpT1 (Figure 2B). The presence of two definable peaks suggests that both species are not interconvertible on the chromatography time scale (<1 h) and implies that dissociation of the dimer is a slow process.

**Figure 3:**
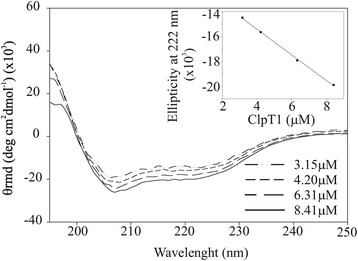
**Effect of protein concentration on ClpT1 conformation.** The far-UV spectrum of ClpT1 was recorded between 190 nm and 250 nm for different protein concentrations. Inset: Correlation between ClpT1 concentration and ellipticity at 222 nm.

Higher order oligomer formation can also be detected by the use of circular dichroism by analyzing molar ellipticity changes with protein concentration [24]. The CD spectra of ClpT1 showed predominant peaks at 208 (π-π* transition) and 222 nm (n-π* transition) (Figure 3) suggesting a high degree of α-helix [25]. The ellipticity value at 208 and 222 nm followed a linear dependence with ClpT1 concentration (Figure 3, inset, only the ellipticity at 222 nm vs. concentration is shown), indicating no relationship between ClpT1 conformation and concentration. This confirmed our previous result that changing ClpT1 concentration does not cause detectable formation of the dimer species. To further support our results that recombinant ClpT1 behaves as a monomer in solution, its hydrodynamic radius (R_h_) was determined by diffusion-ordered spectroscopy (DOSY) (Additional file 1: Figure S1). The R_h_ of a globular protein is directly related to its size, according to the equation R_h_ = 4.75 N^0.29^ Å, where N is the number of amino acids [26]. N for recombinant ClpT1 is 184 so; an R_h_ of 21.6 Å was expected for a monomer. The experimental R_h_ measured by DOSY of ClpT1 at 120 μM was 21.8 Å, in agreement with ClpT1 behaving mainly as a monomer.

**Figure 4:**
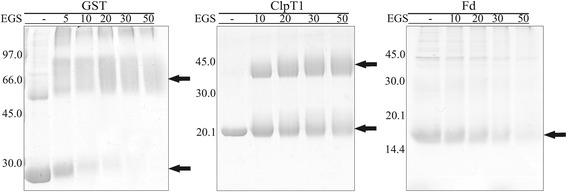
**Crosslinking assays.** Purified proteins were incubated with EGS for 30 min at 25°C. After the treatment, samples were analyzed by SDS-PAGE followed by Coomassie staining. GST and ferredoxin were used as positive and negative controls of oligomerization, respectively. All proteins were at 25 μM, and the fold excess of EGS used in each case is detailed on top. Molecular weights are depicted on the left of each gel.

Our SEC data indicate that dimer formation is a rather weak process. For that reason, cross-linking assays were carried out to stabilize the ClpT1 dimers. As controls, GST (54 kDa dimer protein) [27] and *E. coli* ferredoxin (12 kDa monomeric protein) [28] were used. By this technique, dimer formation was clearly seen, reaching a 60% of the total protein population at the highest cross-linker concentration used (Figure 4). In contrast, dimer formation of GST was complete at a 50-fold molar excess of EGS, while ferredoxin did not assemble into higher order oligomers at any EGS concentration tested.

### Interaction of ClpT1 with Hsp100 chaperones

Specific aspects of the role of the ClpT proteins in the assembly and modulation of the ClpPR proteolytic core is largely unknown, though previous work has shed some light into the problem. By homology modeling, it was proposed that ClpT1/2 dock to the axial pores of the Clp complex, thereby blocking the interaction of ClpPR with the Hsp100 chaperones [15]. Thus, the question that remains is how the ClpT proteins disengage from the complex, allowing the Hsp100 chaperones to interact with it. One possibility we tested is whether the chaperones themselves could aid in this process. First, a possible interaction of ClpT1 with recombinant ClpC2 and ClpD was analyzed by SEC to test whether the migration of ClpT1 through the column was altered. If ClpT1 interacts with the chaperone hexamers, then it would be detected in an elution volume corresponding to a mass range of 500–600 kDa. We have previously used this approach to show the association of ClpC2 hexamers with transit peptide-containing proteins [17]. Yet, no association was found between ClpT1 and ClpC2 or ClpD by this technique (data not shown), as no ClpT1 could be detected in the 500–600 kDa mass range.

**Figure 5:**
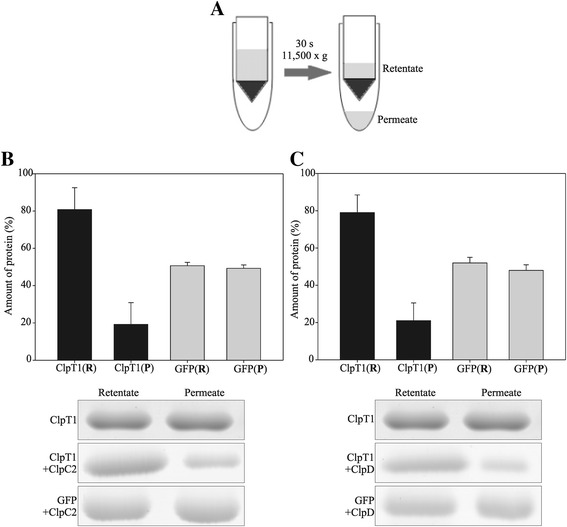
**Interaction of ClpT1 with chloroplastic Hsp100 chaperones from** ***Arabidopsis*** **.** ClpT1 was incubated for 10 min with the chaperones ClpC2 **(B)** and ClpD **(C)** in the presence of 5 mM ATP and subjected to ultrafiltration for 30 s. The experimental setup is shown in **(A)**. The permeate (P) and the retentate (R) were collected and analyzed by SDS-PAGE. Gels were stained with Coomassie Brilliant Blue. GFP was used as a control. The amount of each protein was quantified by gel densitometry using the software GelPro and plotted as a bar chart (standard deviation bars are indicated). Experiments were performed in triplicate; the pictures show a representative result. Bands were cropped from the complete gel image for the sake of clarity. This image is provided in the Additional file 2: Figure S2.

It could be possible that lack of binding was due to fast dissociation of the complex. Then, it could get undetected by SEC since each run is over 1 hour long. For that reason, we established a much faster, ultrafiltration-based strategy (Figure 5A). ClpT1 is a 22 kDa protein; so, when applied to a concentrator equipped with a 50 kDa cut-off membrane, it should pass freely through the membrane and should be detected in the permeate. To test this hypothesis, ClpT1 was subjected to a 30 seconds centrifugation step in a Vivaspin 500 concentrator to allow half of the solution to pass through the membrane. Next, aliquots were taken from the permeate and the retentate and subjected to SDS-PAGE followed by Coomassie staining. The amount of protein present in both fractions was quantified by densitometry of the gels. As expected, approximately 50% of ClpT1 was found in the permeate while the remainder was found in the retentate (Figure 5A and B). This was also true for the green fluorescent protein (GFP, 27 kDa, Additional file 2: Figure S2, Panel D, lanes 6 and 7), which was used as a control. On the contrary, applying ClpC2 or ClpD (93 and 95 kDa, respectively) to the concentrator and using the same centrifugation conditions revealed that both proteins were completely retained [i.e. they did not pass through the membrane, Additional file 2: Figure S2, lanes 8 and 9 in Panel C (ClpC2) and D (ClpD)]. In another set of experiments, ClpT1 was applied to the concentrator in the presence of either ClpC2 or ClpD and 5 mM MgATP and centrifuged briefly as before. Under these conditions, ClpT1 was retained in the retentate containing the chaperones by more than 75% (Figure 5B and C), indicating a physical interaction with the Hsp100 proteins. On the other hand, subjecting GFP to the same experimental setup did not alter its migration through the membrane, which shows that retention of ClpT1 by the chaperones was protein specific. In some cases, protein precipitation or aggregation can occur during ultrafiltration. To exclude that these processes were not the reason for the retention of ClpT1, retentates were subjected to centrifugation followed by SEC. In all cases, ClpT1 and the Hsp100 chaperones remained soluble and maintained their migration profile after the ultrafiltration experiments (Additional file 3: Figure S3).

### Hsp100 ATPase activity modulation by ClpT1

**Figure 6:**
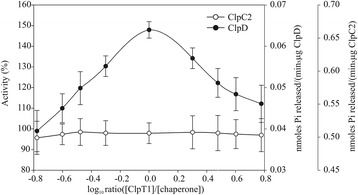
**Influence of ClpT1 in the ATPase activity of ClpC2 and ClpD.** Release of inorganic phosphate was monitored spectrophotometrically by the Malachite green method. The% activity of each chaperone in the presence of varying amounts of ClpT1 was calculated as [specific activity (nmoles inorganic phosphate/minxμg protein) in the presence of ClpT1 x 100/specific activity in the absence of ClpT1]. The% activity is plotted as a function of the log[ClpT1]/[Hsp100] molar ratio. The additional axes on the right show the scale of specific activity of ClpC2 and ClpD. The experiments were performed in triplicate.

As ClpT1 associated with ClpC2 and ClpD, we tested if it could also modify their ATPase activity. Both chaperones have a basal ATPase activity that can be followed by the Malachite green method. ClpT1 was incubated with either chaperone in different molar ratios ranging from approximately 0.15:1 to 6:1 (ClpT1:chaperone). The ATPase activity of ClpC2 was not altered by the presence of ClpT1 at any concentration tested. However, the ATPase activity of ClpD was activated in a concentration dependent manner, reaching a maximal activation of >50% at a 1:1 molar ratio (Figure 6). Interestingly, ATPase activity did not plateau after this point but decreased at higher molar excess of ClpT1, lowering to the basal value at a 6:1 ratio.

The K_m_ and V_max_ of ClpD in the absence and presence of ClpT1 at a 1:1 molar ratio were determined. As we previously observed, the ATPase activity of ClpD did not reach complete saturation within the ATP concentration range used for the analysis [17]. The kinetic parameters were estimated by fitting the data points (Additional file 4: Figure S4). The K_m_ and V_max_ of ClpD in the absence of ClpT1 were 19.1 mM and 0.19 nmol/(min × μg protein), and those of ClpD in the presence of ClpT1 at a 1:1 molar ratio (maximal activation) were 28.3 mM and 0.43 nmol/(min × μg protein) respectively. The data reveals that ClpT1 induced an increase on the V_max_ and the K_m_, albeit to a lesser extent. This observation can be taken as an indication that ClpT1 is producing some structural variation on ClpD, which may change the energy barriers that govern the rate of ATP hydrolysis. An uncoupling between ATP hydrolysis and the chaperone function (i.e., the polypeptide threading or pulling) may account for that change.

## Discussion

The chloroplastic Clp protease has been regarded as a constitutive housekeeping enzyme [29]. As such, the protein levels of its constituents remain constant in both normal and stressful conditions [30]. However, the needs for proteolysis are not expected to be the same under different situations. This led to the current notion that proteolytic activity of the Clp protease seems to be regulated by substrate recognition mechanisms and interaction with accessory proteins and chaperones, namely ClpT (1 and 2), ClpS and the Hsp100 chaperones [31,32].

Two non-mutually exclusive models have been proposed that attempt to explain the role of ClpTs. Sjögren and Clarke found that the ClpT proteins are involved in the assembly of the Clp protease. They suggested that almost all of ClpTs exist as homogeneous dimers in the stroma and, after monomerization by an unknown mechanism, the monomers bind to P-rings with high affinity. ClpT1/2-loaded P-rings then associate with R-rings to form the Clp core complex [21]. On the other hand, Peltier et al. modeled the structure of ClpT1 using a three-dimensional threading tool [15]. For this, the N-terminal *E. coli* ClpA domain was used as the template as it shows a notable sequence-to-structure alignment with ClpTs. Neighboring ClpP proteins in the P-ring form hydrophobic pockets that display remarkable complementarities in shape and hydrophobicity/polarity with loops present in the ClpT proteins. By rigid docking of the backbones of ClpT1 and ClpP3 and 6, Peltier et al. placed the ClpT proteins near the axial openings of the Clp peptidase, though which the substrates enter into the central chamber. The Hsp100 chaperones are also proposed to bind to the apical side of the peptidase, thus acting as entrance gates for unfolded polypeptides. As a result, the binding of ClpT1/2 would directly compete with the association of the hexameric Clp chaperones to the protease. In this situation, the role of ClpT1/2 would be to modulate Hsp100 chaperone docking and substrate delivery. This model was later revised by the same group in light of new experimental data. As explained, the plastid ClpP/R protease complex in *Arabidopsis* is asymmetrical, as it is made of two rings with different subunit composition. Olinares et al. suggested that the ClpP1/R ring is the docking site for Hsp100 chaperones [33] while Sjögren and Clarke showed that ClpTs only interact with the ClpP3-P6 ring [21]. Under this model, it is unclear why the Hsp100 chaperones should displace ClpT1 and T2 at all, since their docking sites are opposite to one another. However, it should also be noted that there is no experimental evidence that the Hsp100 chaperones bind only to one side of the Clp protease. In *E. coli*, it is clear that ClpA binds to both sides of the ClpP tetradecamer [34]. In this case, binding of ClpT proteins to the axial pores of the Clp protease would interfere with Hsp100 association and their removal would lead to the completion of the Clp(C/D)/ClpPR degradation machine, as proposed in the earlier model of Peltier et al.

Our results add new evidence to the functioning of the Clp complex but also call into question some aspects. First of all, we could not detect large amounts of ClpT1 dimers by several techniques under various experimental conditions. Significant amounts of the dimer species was only seen by stabilization with a crosslinker reagent, suggesting a weak association. Dimer dissociation and subsequent availability of free monomers is a key step in the model of Sjögren and Clarke. In their report, recombinant and native ClpT1 dimers were detected in native gradient PAGE gels after 48 hs of electrophoresis. In this technique though, molecular mass determinations in extended runs were shown to deviate from real values, especially for proteins with molecular mass below 100 kDa [35,36]. In addition, given the strong sequence-to-structure alignment of ClpT1 with the N-terminus of ClpA, similarity in some biophysical characteristics can be expected. Lo and coworkers showed that the N-terminal repeat domain of *E. coli* ClpA (residues 1–161, same residues used by Peltier et al. for homology modeling) (i) has a CD spectrum very similar to the one we obtained for ClpT1 and (ii) behaved as a monomer in analytical equilibrium ultracentrifugation experiments [37]. Taking our data into account, dimerization of ClpTs can be confirmed, yet more experimental evidence is needed to establish the true conformation in the stroma and the kinetics of dimer formation and their stability. It is important to keep in mind that we have used recombinant ClpT1. It could be possible that recombinant ClpT proteins differ from the native ones in their ability to oligomerize, which was also noticed by Sjögren and Clarke. If in fact ClpT1 forms stable dimers in the stroma, then dissociation by Hsp100 chaperones could be the mechanism of monomerization, a phenomenon we cannot test with recombinant ClpT1. Alternatively, since ClpT1 and ClpT2 are involved in the assembly of the Clp protease, then their displacement by Hsp100 chaperones could lead to the disassembly of the complex, a point that has not been addressed so far. It can be proposed that when proteolysis is not longer needed and a substrate has been fully processed, the Hsp100 chaperones disengage from the core protease and remove ClpT1 and T2 from the core, leading to its disassembly and inactivation. Interestingly, the amount of stromal Clp proteolytic core increases 2.5 times in a *clpC1* mutant [38], which is line with our hypothesis.

In any case, a direct physical interaction between the ClpT proteins and the Hsp100 chaperones is necessary. Hsp100 chaperones have protein remodeling activities; i.e., the ability to change the biological activity of a protein complex by modifying its structure [39,40]. We speculate that chloroplastic Hsp100 chaperones may exert this ability in order to remodel the ClpT proteins. The results from the ultrafiltration assays indicate that the chaperones can specifically interact with ClpT1. The same experimental approach was used to demonstrate the remodeling activity of *E. coli* ClpA on RepA, the initiator protein of the P1 plasmid [41]. Oligomer dissociation of RepA by ClpA is an ATP-dependent mechanism. In ATPase activity assays, ClpD ATPase activity was increased by more than 50% with the addition of an equimolar amount of ClpT1. The shape of the activation curve is somewhat puzzling. If ClpT1 acted as a substrate for ClpD, then a hyperbolic curve would be expected. The obtained bell-shaped curve indicates that maximal activation occurs at a 1:1 molar ratio, but excess ClpT1 somehow inhibits the increase in ATPase activity. This suggests that excess ClpT1 acts at a regulatory site, modulating ClpD ATPase activity tightly. ClpT1 at a 1:1 molar ratio may uncouple the ATPase activity of ClpD from its ability to force polypeptides to the proteolytic core, which explains the increase in the kinetic parameters of the chaperone. In a previous study, we show that ClpD possesses a much lower intrinsic ATPase activity than ClpC2 [17], so an activity increase for protein remodeling may be necessary only for ClpD. This may explain why ClpC2 ATPase activity was not activated by ClpT1, even though a physical interaction does occur.

The findings presented in this work reassure the notion that the activity of the Hsp100/Clp complex is regulated by means other than differential regulation of *clp* gene expression. Many examples in other organisms indicate that interaction with accessory proteins serve this purpose. In bacteria, ClpS binds to ClpA reducing its affinity for unfolded polypeptides [42]. In *Bacillus subtilis*, the chaperone activity of ClpC is modulated by the adaptor protein MecA [43]. In addition, NblA is an adaptor protein that binds to ClpC, bringing it to a close contact with phycobiliproteins [44]. This interaction is needed for proteolytic degradation of phycobilisomes in cyanobacteria. With our discovery of the interplay between ClpT1 and the Hsp100 chaperones, a new layer of regulation is introduced. Further biochemical analyses will be needed to establish the mode of action of accessory proteins of the Hsp100/Clp complex.

## Conclusions

We have purified the *A. thaliana* chloroplast protein ClpT1 and demonstrated that it interacts with the Hsp100 chaperones ClpC2 and ClpD and modulates the ATPase activity of the latter. A thorough analysis of its oligomerization status *in vitro* showed that monomers are many times more abundant than dimers. The findings provide new insights into the role of this accessory protein in the regulation of the activity of the Hsp100/Clp protease complex.

## Methods

### Plasmid construction

ClpT1 cDNA was obtained from the RIKEN cDNA bank (pda: 02480). The cDNA region coding for the mature protein was amplified using Platinum Pfx DNA polymerase (Life Technologies). The primers contained restriction sites for directional cloning in plasmid pET28a(+) (Novagen): 5′- GGT*CCATGG*CCTCGGCCAGCACGG -3′; and 5′- GAAG*CTCGAG*GCTGCCGCGCGGCACCAG*GAATTC*TTGACCTTGTTTCTTGAAGCTC -3′ (restriction sites for NcoI, EcoRI, and XhoI respectively, are in italics). In this construction, the protein is produced as a fusion to a C-terminal hexahistidine tag with a thrombin cleavage site between the protein and the tag. The final construct was checked by DNA sequencing.

### Expression and purification of ClpT1

The resulting plasmid was transformed into the *E. coli* BL21(DE3) Codon Plus-RIL strain (Novagen). The cells were grown in 1 L of Terrific Broth media at 37°C until an A_600_ of 0.6-0.7 was reached. The temperature was lowered to 25°C and the inducer isopropyl-beta-D-thiogalactopyranoside (IPTG) was added to a final concentration of 0.5 mM. After six hours of induction, cells were harvested by centrifugation and resuspended in cold lysis buffer (50 mM Tris–HCl pH 8.0, 400 mM NaCl, 1 mM benzamidine, 10%v/v glycerol) at a 25:1 ratio (mL culture:mL buffer). The cells were lysed by two passages through a French Press (Aminco) and the soluble fraction was recovered by centrifugation (30,000 × g, 1 h). The supernatant was supplemented with 500 μL of Ni^2+^-NTA-agarose resin (Qiagen) and incubated for 1 h. The mixture was transferred to a column and washed with 30 column volumes of lysis buffer supplemented with 20 mM imidazole. Lysis buffer plus 250 mM of imidazole was used to elute the recombinant protein in 100 μL fractions. These fractions were desalted by dialysis using a 12,000 Da cut-off membrane against dialysis buffer (50 mM Tris–HCl pH 8.0, 100 mM NaCl, 10%v/v glycerol) for 16 h. To remove the polyhistidine tag, 1 mg of recombinant protein was incubated in the presence of 3 units of thrombin at 10°C for 16 h. The preparations were then loaded onto a Ni^2+^-NTA-agarose column to remove free tags and undigested protein. A final step consisting of a passage through a Sephadex-75 SEC column (described below) was necessary to reach a purity level of at least 98%, as assayed by Coomassie-stained 12% SDS-PAGE gels. Molecular weight markers were from GE (Low molecular weight calibration kit for SDS electrophoresis). The proteins ClpC2 and ClpD were purified as described previously [17]. Protein concentration was determined by the Bradford method using BSA as standard protein [45].

### Circular dichroism assays

The purified protein was equilibrated in 10 mM phosphate buffer (pH 7.44). CD experiments in the far-UV region (195–250 nm) were carried out using a 1 mm path-length quartz cuvette at 25°C in a Jasco J-810 spectropolarimeter equipped with a Peltier temperature-controlled cell holder (Easton). The instrument was purged with a continuous flow of nitrogen at 5 L/min. Spectra obtained in the far-UV are presented without mathematical smoothing. The informed spectrum is the average from 10 spectra, each measured at a scan rate of 1 nm/s. For oligomerization analysis, the mean residue molar elipticity at 222 nm was plotted against the concentration of ClpT1.

### Size exclusion chromatography

Purified samples were loaded onto a Superdex 75 10/300 GL column (GE) attached to an Äkta Prime chromatography system. The runs were performed at a flow rate of 0.5 mL/min using a degassed buffer made of 50 mM Tris–HCl pH 8.0, 100 mM NaCl. Molecular weight standards were used to calibrate the column (MWGF1000 kit for molecular weights 29,000–700,000 and apronitin, Sigma-Aldrich). The molecular weight of the protein ClpT1 was also determined on a Precision Detectors PD2010 light scattering instrument connected *in tandem* to a high-performance liquid chromatography system as described in [46].

### Hydrodynamic radius determination

Diffusion-ordered spectroscopy experiments were acquired at 25°C on a Bruker Avance II 600 MHz spectrometer using a triple-resonance probe equipped with z-axis self-shielded gradient coils. ClpT1 (120 μM) was dissolved in 10 mM phosphate buffer pH 7.4 in D_2_O and containing dioxane as an internal radius standard (2.12 Å) and viscosity probe. The gradient strength was shifted from 0.68 to 32.35 G/cm in a linear manner. Acquisition, processing, and visualization of the spectra were performed using TOPSPIN 2.1 (Bruker) and Sparky.

### Cross-linking assays

The cross-linker ethylene glycolbis(succinimidylsuccinate) (EGS) was dissolved in dimethyl sulfoxide at a concentration of 25 mM. Cross-linking reactions were carried out in a reaction mixture containing 50 mM Hepes pH 7.5, 100 mM NaCl and 25 μm of the corresponding protein. As controls of dimeric and monomeric proteins, glutathione *S*-transferase (GST) and *E. coli* ferredoxin were used respectively. EGS was added to the reaction mixture at different molar proportions: 10, 20, 30 and 50-fold molar excess. The reactions were incubated for 30 min at room temperature. Then, the EGS was quenched by the addition of 50 mM Tris pH 7.5. Samples were subjected to 12% SDS-PAGE as described elsewhere.

### Ultrafiltration assays

In a buffer containing 50 mM Tris–HCl pH 8.0, 150 mM NaCl, 10 mM MgCl_2_ and 5 mM ATP, ClpC2 or ClpD and ClpT1 or GFP (negative control) were coincubated at a final concentration of 1.3 μM each in a final volume of 150 μL. After incubation for 10 min at 25°C, the mixture was centrifuged at 11,500 × g in a Vivaspin 500 centrifugal concentrator with a 50,000 Da cut-off membrane (GE). Centrifugation time was limited to 30 s to allow half of the solution to pass through the membrane. The permeate and the remaining solution (retentate) were collected and loaded in SDS-PAGE gels. The amount of ClpT1 or GFP in each fraction was measured by densitometry of the bands. Experiments were done in triplicates.

### ATPase activity assays

The ATPase activity of ClpC2 and ClpD was measured by the release of inorganic phosphate using the Green malachite method as previously described [47]. In the assays, the concentration of the Hsp100 chaperones was 0.5 μM and the concentration of ClpT1 was varied from 0.08 to 3.15 μM so that the log [molar ratio (ClpT1/Hsp100)] varied from −0.8 to 0.8. For the determination of kinetic parameters, the concentration of ClpD and ClpT1 was 0.5 μM, and the ATP concentration was varied from 0 to 12 mM. Experiments were done in triplicates.

## Additional files

Additional file 1: Figure S1. ClpT1 hydrodynamic radius determination by DOSY. The file contains a plot of the DOSY signal intensity of ClpT1 (A) and dioxane (B) as a function of gradient strength. Decay rates were calculated by fitting each curve and the hydrodynamic radius was determined as described in [26]. Additional file 2: Figure S2. Distribution of Hsp100 chaperones, ClpT1 and GFP (alone or in combination) in ultrafiltration assays. The file contains an analysis of the distribution of ClpC2, ClpD and GFP in the absence of ClpT1 in ultrafiltration assays. This analysis serves as a control of the assays described in the main body text. The file also contains the complete gel images from which the data for Figure 5 was taken. Additional file 3: Figure S3. SEC analysis of ClpC2/ClpT1 and ClpD/ClpT1 mixtures after ultrafiltration. After ultrafiltration experiments of ClpT1 in the presence of ClpC2 or ClpD, the retentates were subjected to SEC, using a Superdex 75 column as previously described. Elution profiles of ClpT1 alone and in the presence of ClpC2 or ClpD are shown. Additional file 4: Figure S4. Kinetic analysis of ClpD ATPase activity in absence and presence of ClpT1 in a 1:1 molar relationship. The specific ATPase activity of ClpD is represented as a function of ATP concentration, in the absence and presence of 0.5 μM ClpT1. Data points represent the mean of triplicate experiments, the standard error remained below 15% in every condition. The curves were fitted to the Michaelis-Menten equation (strong lines) using Sigma Plot.
