# Supplementary material for: Characterization of the accessory protein ClpT1 from Arabidopsis thaliana: oligomerization status and interaction with Hsp100 chaperones
Source: BMC Plant Biol. 2014 Aug 24;14:228. doi: 10.1186/s12870-014-0228-0 (PMC4243950; doi:10.1186/s12870-014-0228-0)
Supplement: Additional file 1: Figure S1. — ClpT1 hydrodynamic radius determination by DOSY. The file contains a plot of the DOSY signal intensity of ClpT1 (A) and dioxane (B) as a function of gradient strength. Decay rates were calculated by fitting each curve and the hydrodynamic radius was determined as described in [26]. [file 12870_2014_228_MOESM1_ESM.pdf]

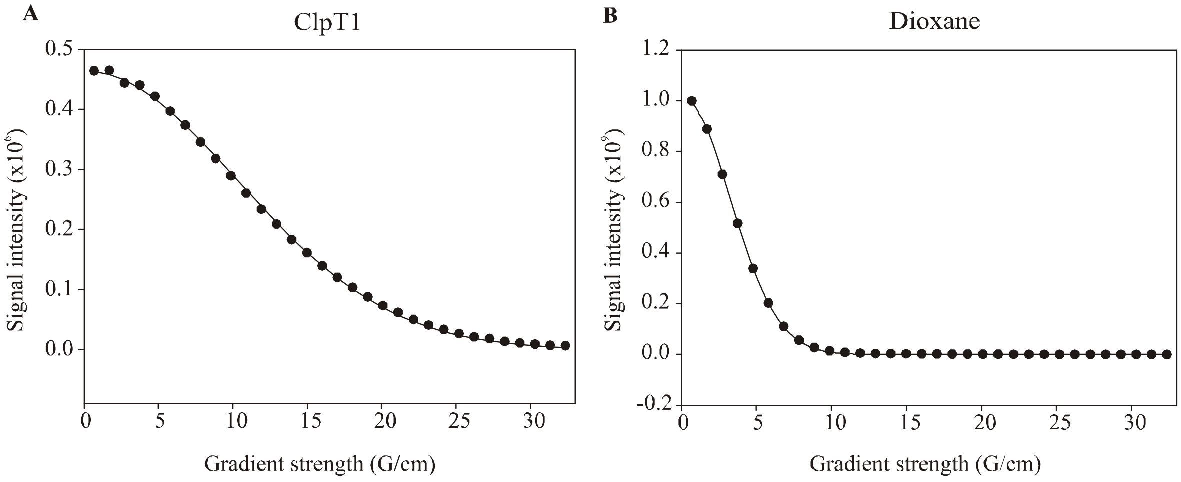

Additional Figure 1: ClpT1 hydrodynamic radius determination by DOSY. The plot of DOSY signal intensity of ClpT1 (A) and dioxane (B) as a function of gradient strength is shown. Decay rates were calculated by fitting each curve and the hydrodynamic radius was determined as described in [26].
