# Supplementary material for: Characterization of the accessory protein ClpT1 from Arabidopsis thaliana: oligomerization status and interaction with Hsp100 chaperones
Source: BMC Plant Biol. 2014 Aug 24;14:228. doi: 10.1186/s12870-014-0228-0 (PMC4243950; doi:10.1186/s12870-014-0228-0)
Supplement: Additional file 2: Figure S2. — Distribution of Hsp100 chaperones, ClpT1 and GFP (alone or in combination) in ultrafiltration assays. The file contains an analysis of the distribution of ClpC2, ClpD and GFP in the absence of ClpT1 in ultrafiltration assays. This analysis serves as a control of the assays described in the main body text. The file also contains the complete gel images from which the data for Figure 5 was taken. [file 12870_2014_228_MOESM2_ESM.pdf]

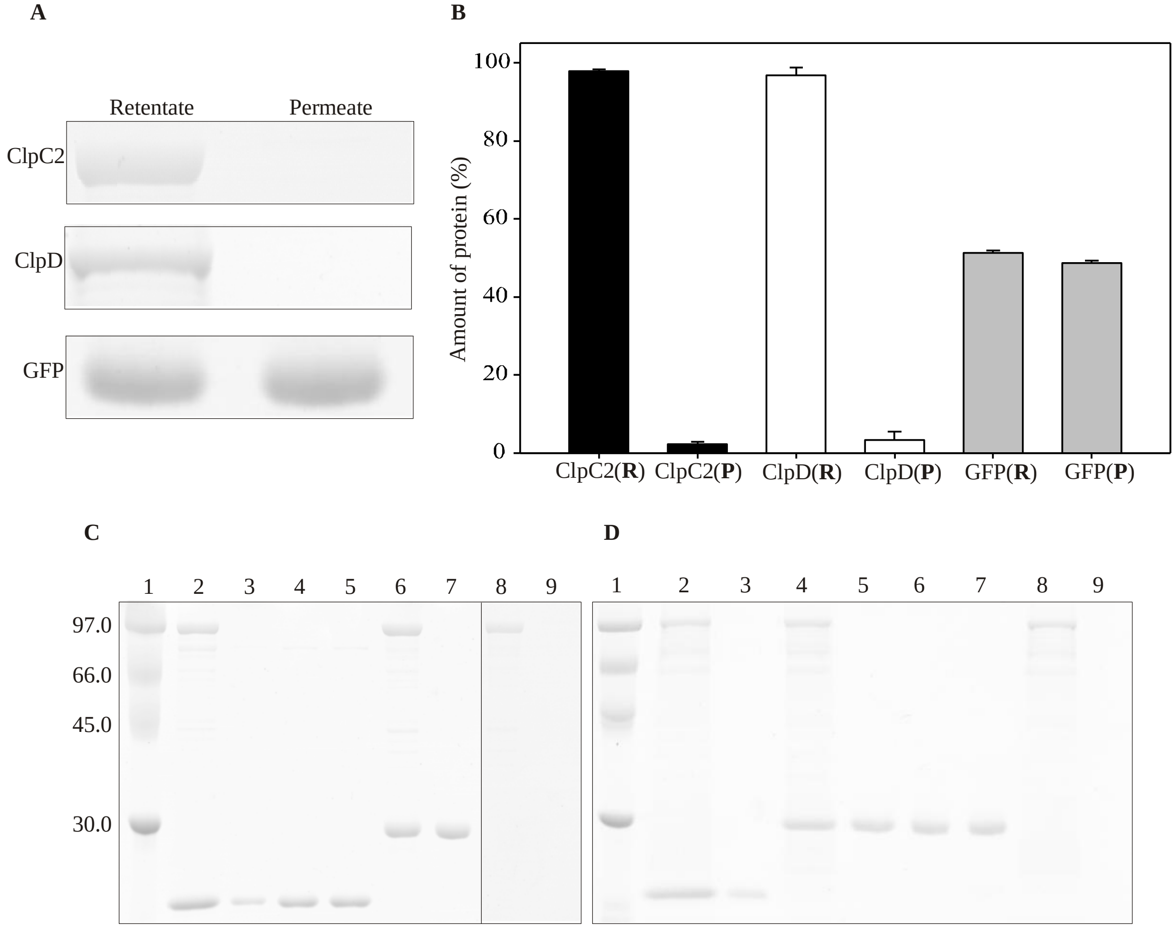

Additional Figure 2. Distribution of Hsp100 chaperones, ClpT1 and GFP (alone or in combination) in ultrafiltration assays. (A) The chaperones ClpC2, ClpD and the control protein GFP were subjected to ultrafiltration in the conditions described in the Methods section in the absence of other proteins. The permeate and the retentate were collected and analyzed by SDS-PAGE and Coomassie staining. Bands were cropped from the gel images displayed in Panels C and D: lanes 8 and 9 from Panel C for ClpC2, lanes 8 and 9 from Panel D for ClpD and lanes 6 and 7 from Panel D for GFP. (B) The amount of protein in each band was quantified by densitometry using the software GelPro and plotted as a bar chart

(standard deviation bars are indicated), **P**: permeate, **R**: retentate. Experiments were performed in triplicate. (C) Complete image of the SDS-PAGE analysis of the interaction of ClpT1 with ClpC2, from which the data displayed in Figure 5 was taken. Lane 1: molecular weight marker, lane 2: ClpC2 + ClpT1 (**R**), lane 3: ClpC2 + ClpT1 (**P**), lane 4: ClpT1 (**P**), lane 5: ClpT1 (**R**), lane 6: ClpC2 + GFP (**R**), lane 7: ClpC2 + GFP (**P**), lane 8: ClpC2 (**P**), lane 9: ClpC2 (**R**). These last two lanes were cropped from a different gel. (D) Complete image of the SDS-PAGE analysis of the interaction of ClpT1 with ClpD, from which the data displayed in Figure 5 was taken. Lane 1: molecular weight marker, lane 2: ClpD + ClpT1 (**R**), lane 3: ClpD + ClpT1 (**P**), lane 4: ClpD + GFP (**R**), lane 5: ClpD + GFP (**P**), lane 6: GFP (**P**), lane 7: GFP (**R**), lane 8: ClpD (**P**), lane 9: ClpD (**R**).
