# Supplementary material for: Characterization of the accessory protein ClpT1 from Arabidopsis thaliana: oligomerization status and interaction with Hsp100 chaperones
Source: BMC Plant Biol. 2014 Aug 24;14:228. doi: 10.1186/s12870-014-0228-0 (PMC4243950; doi:10.1186/s12870-014-0228-0)
Supplement: Additional file 3: Figure S3. — SEC analysis of ClpC2/ClpT1 and ClpD/ClpT1 mixtures after ultrafiltration. After ultrafiltration experiments of ClpT1 in the presence of ClpC2 or ClpD, the retentates were subjected to SEC, using a Superdex 75 column as previously described. Elution profiles of ClpT1 alone and in the presence of ClpC2 or ClpD are shown. [file 12870_2014_228_MOESM3_ESM.pdf]

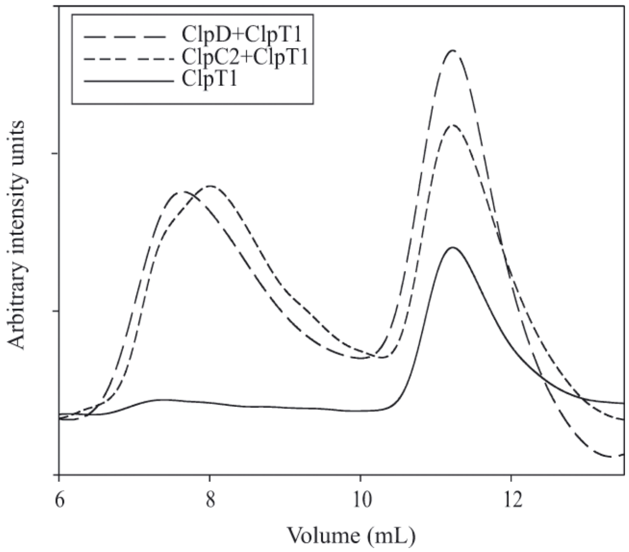

Additional Figure 3: SEC analysis of ClpC2/ClpT1 and ClpD/ClpT1 mixtures after ultrafiltration. After ultrafiltration experiments of ClpT1 in the absence and presence of ClpC2 or ClpD, the retentates were subjected to SEC, using a Superdex 75 column as previously described. Elution profiles of ClpT1 alone and in the presence of ClpC2 or ClpD are shown. ClpD eluted at 7.65 mL and ClpC2 at 8.00 mL ClpT1 migrated at 11.24 mL, as previously shown in Figure 2.
