# Supplementary material for: Characterization of the accessory protein ClpT1 from Arabidopsis thaliana: oligomerization status and interaction with Hsp100 chaperones
Source: BMC Plant Biol. 2014 Aug 24;14:228. doi: 10.1186/s12870-014-0228-0 (PMC4243950; doi:10.1186/s12870-014-0228-0)
Supplement: Additional file 4: Figure S4. — Kinetic analysis of ClpD ATPase activity in absence and presence of ClpT1 in a 1:1 molar relationship. The specific ATPase activity of ClpD is represented as a function of ATP concentration, in the absence and presence of 0.5 μM ClpT1. Data points represent the mean of triplicate experiments, the standard error remained below 15% in every condition. The curves were fitted to the Michaelis-Menten equation (strong lines) using Sigma Plot. [file 12870_2014_228_MOESM4_ESM.pdf]

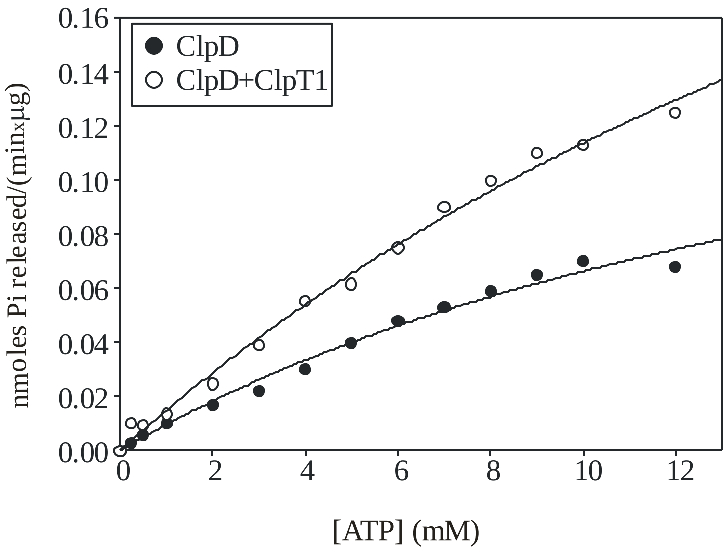


**Additional Figure 4: Kinetic analysis of ClpD ATPase activity in absence and presence of ClpT1 in a 1:1 molar relationship.** The specific ATPase activity of ClpD is represented as a function of ATP concentration, in the absence and presence of 0.5 µM ClpT1. Data points represent the mean of triplicate experiments, the standard error remained below 15% in every condition. The curves were fitted to the Michaelis-Menten equation (strong lines) using Sigma Plot.
